# Unique Features of River Lamprey (*Lampetra fluviatilis*) Myogenesis

**DOI:** 10.3390/ijms23158595

**Published:** 2022-08-02

**Authors:** Marta Migocka-Patrzałek, Roman Kujawa, Piotr Podlasz, Dorota Juchno, Katarzyna Haczkiewicz-Leśniak, Małgorzata Daczewska

**Affiliations:** 1 Department of Animal Developmental Biology, Faculty of Biological Sciences, University of Wrocław, Sienkiewicza 21, 50-335 Wroclaw, Poland; 2 Department of Ichthyology and Aquaculture, Faculty of Animal Bioengineering, University of Warmia and Mazury in Olsztyn, Oczapowskiego 2, 10-719 Olsztyn, Poland; reofish@uwm.edu.pl; 3 Department of Pathophysiology, Forensic Veterinary Medicine and Administration, Faculty of Veterinary Medicine, University of Warmia and Mazury in Olsztyn, Oczapowskiego 13, 10-719 Olsztyn, Poland; piotr.podlasz@uwm.edu.pl; 4 Department of Zoology, Faculty of Biology and Biotechnology, University of Warmia and Mazury in Olsztyn, Oczapowskiego 5, 10-719 Olsztyn, Poland; juchno@uwm.edu.pl; 5 Division of Ultrastructural Research, Faculty of Medicine, Wroclaw Medical University, Chalubinskiego 6a, 50-368 Wroclaw, Poland; katarzyna.haczkiewicz@umw.edu.pl

**Keywords:** river lamprey, *Lampetra fluviatilis*, myogenesis, MfPax3/7, Mrf5, myogenic factors, somitogenesis, larval musculature, embryogenesis

## Abstract

The river lamprey (*L. fluviatilis*) is a representative of the ancestral jawless vertebrate group. We performed a histological analysis of trunk muscle fiber differentiation during embryonal, larval, and adult musculature development in this previously unstudied species. Investigation using light, transmission electron (TEM), and confocal microscopy revealed that embryonal and larval musculature differs from adult muscle mass. Here, we present the morphological analysis of *L. fluviatilis* myogenesis, from unsegmented mesoderm through somite formation, and their differentiation into multinucleated muscle lamellae. Our analysis also revealed the presence of myogenic factors LfPax3/7 and Myf5 in the dermomyotome. In the next stages of development, two types of muscle lamellae can be distinguished: central surrounded by parietal. This pattern is maintained until adulthood, when parietal muscle fibers surround the central muscles on both sides. The two types show different morphological characteristics. Although lampreys are phylogenetically distant from jawed vertebrates, somite morphology, especially dermomyotome function, shows similarity. Here we demonstrate that somitogenesis is a conservative process among all vertebrates. We conclude that river lamprey myogenesis shares features with both ancestral and higher vertebrates.

## 1. Introduction

The river lamprey (*Lampetra fluviatilis*) is a representative of the ancestral, jawless vertebrate group. This species is extremely interesting in terms of its biology in evolutionary and phylogenetic aspects. During the lamprey’s life cycle, four phases can be distinguished: embryonic, larval, juvenile, and adult. The characteristic features of the lamprey life cycle are a long-lasting larval stage (3–6 years), metamorphosis, and relatively short adulthood with distinctly different anatomy and physiology. After metamorphosis, adult river lampreys migrate downriver to the sea, where they lead a parasitic lifestyle, feeding on fish. In spring, sexually mature individuals return to freshwater spawning grounds, where they die after spawning [1,2]. Lamprey metamorphosis, regulated like amphibians by thyroid hormone, is characterized by major changes in their anatomy and physiology. It includes changes in the gonads, digestive system, liver, and kidneys, loss of the oral hood and its replacement with a multi-toothed oral disc, formation of well-developed eyes, and development of larger dorsal fins. Changes also occur in the skeletal muscle structure [1,3].

It has been demonstrated that the lamprey trunk musculature, as in all jawed (Gnathostomata) vertebrates, derives from myotomes [4]. In all jawed vertebrates, multinucleated muscle fibers are derived from the paraxial mesoderm, which is divided into segmental blocks of cells called somites. During embryogenesis, somites differentiate into the epithelial dermomyotome, the myotome, and the mesenchymal sclerotome (Stern et al., 1995). The dermomyotome is the main source of skin connective tissue and skeletal muscles (e.g., trunk and limb), whereas the sclerotome is a source of material for the axial skeleton. The dermomyotome consists of dorsal medial and ventrolateral lips (DML and VLL, respectively). It has been shown that the dermomyotome is a source of muscle progenitor cells (satellite cells, differentiate to the myoblasts), which are Pax3- and Pax7-positive (paired box-transcription factor Pax3 and Pax7 proteins) [5,6,7,8,9]. Muscle progenitor cells migrate from the dermomyotome and initially fuse to form multinucleated myotubes (immature muscle fibers) and then differentiate into muscle fibers [10]. Comparative studies on trunk muscle differentiation in vertebrates revealed that although in all adult individuals muscle fibers are multinucleated, the pathway leading to multinuclearity differs among species belonging to different vertebrate taxa. In fish, such as sturgeons and lungfish, during early myogenesis, myoblasts differentiate into multinucleated lamellae or, as in teleost fish, into multinucleate myotubes [11,12,13,14,15,16]. In amphibians, myoblasts fuse to form multinucleated myotubes (e.g., *Triturus vulgaris*, *Rana* *lessonae*) or, bypassing fusion, directly differentiate into mononucleated myotubes (e.g., *Xenopus* *laevis*, *Hymenochirus boettgeri*, *Bombina variegata*) [17,18,19,20,21,22]. Mononucleated myotubes were also observed during primary myogenesis in amniotes [5,23,24,25,26].

Myogenesis in vertebrates is under the control of a conservative myogenic program governed by the Pax3/Pax7 and myogenic regulatory factor (MRF) network. MRFs consist of four main transcription factors: MyoD, Myf5, myogenin, and MRF4 [27,28,29,30,31]. These factors regulate muscle fiber differentiation at various stages of myogenesis. Expression of MyoD and Myf5 determines entry of undifferentiated cells into the myogenic program, whereas myogenin and MRF4 control myotube maturation and differentiation into muscle fibers (reviewed by Buckingham [32]).

The jawed vertebrate’s trunk skeletal muscles consist of epaxial (dorsal) and hypaxial (ventral) portions, separated by the horizontal myoseptum [5,6]. In fish, whose musculature is best anatomically visible, in the myotomes, peripherally situated red (slow), centrally located white (fast), and between them, pink muscles, can be distinguished. The muscle types differ from each other structurally and physiologically. Compared to white muscles, red muscles are rich in dense capillaries, myoglobin, and mitochondria. They can contract slowly for a long period without fatigue. On the other hand, white muscles can contract quickly with great force but not for very long. In white muscle fibers, the contractile apparatus completely fills the sarcoplasm, in contrast to red muscle fibers, where it occupies only part of the sarcoplasm. Pink fibers share anatomical and physiological features of both red and white muscles [33].

In our study, we performed a histological analysis of trunk muscle fiber differentiation during embryonal, larval, and adult musculature development in a previously unstudied species, the river lamprey (*L.* *fluviatilis*). Using light, transmission electron (TEM), and confocal microscopy, we discovered that embryonal and larval musculature differs from adult muscle mass. During the early steps of myogenesis, myoblasts fuse to form multinucleated muscle lamellae, whereas in adults, cylindrical multinucleated muscle fibers are observed. The process of muscle fiber formation in adults is not known. Since the larval period in river lamprey is relatively long, difficulties arise in obtaining individuals during metamorphosis, when the process of muscle lamellae disappearance and formation of adult muscle fibers occurs.

## 2. Results

### 2.1. Embryonal and Larval Myogenesis

**Figure 1:**
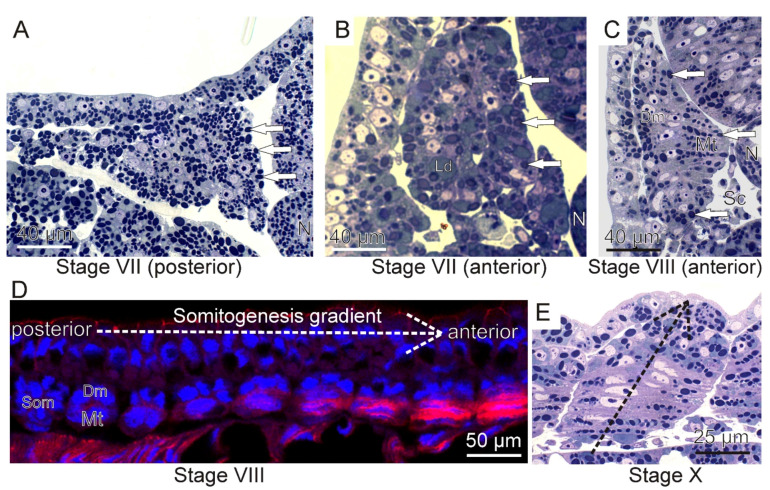
**Early stages of river lamprey (*L. fluviatilis*) embryogenesis.** Cross (**A**–**C**) and longitudinal (**E**) sections of trunk muscles are stained with methylene blue. (**D**) Longitudinal confocal section of trunk muscles. Nuclei (blue), actin (red). Yolk platelets (white arrows), notochord (N), lipid droplets (Ld), dermomyotome (Dm), myotome (Mt), sclerotome (Sc), somite (Som). The white dashed arrow shows a posterior–anterior gradient of somitogenesis. The black dashed arrow shows the mediolateral gradient of myoblast fusion in the myotome.

During the early stages of river lamprey embryogenesis (stage VII), the unsegmented mesoderm (paraxial) is located on both sides of the neural tube and notochord under the single layer of epithelium covering the whole embryo. The cells of the paraxial mesoderm are oval and rich in yolk platelets (Figure 1A).

**Figure 2:**
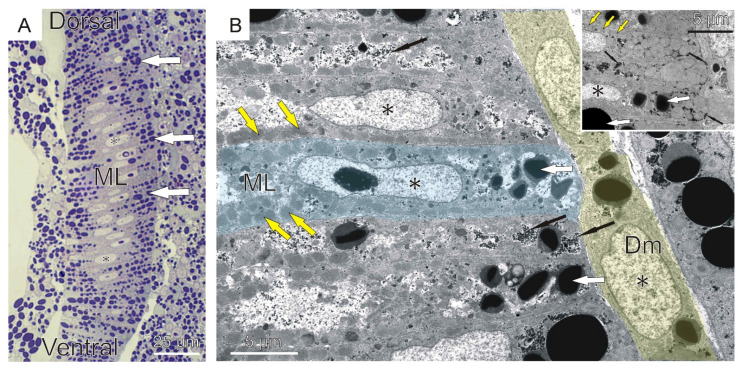
**Formation of multinucleated muscle lamellae during river lamprey (*L. fluviatilis*) myogenesis (stage X).** (**A**) Cross section through the trunk muscles stained with methylene blue. (**B**) Ultrastructure of the embryo skeletal muscles. Inset—terminal parts of muscle lamellae. Muscle lamellae (ML, colored in blue at (**B**)), yolk platelets (white arrows), dermomyotome (Dm, colored in yellow), myofibrils (yellow arrows), glycogen granules (black, thin arrows), nuclei (*).

As a result of somitogenesis, blocks of cells, called somites, separate from the unsegmented mesoderm. Somites, as metameric units, similar to the structures present in other jawed vertebrates, are located in the dorsal region of the embryo on either side of the notochord and the neural tube. The early somite wall is built of a single layer of elongated epithelial cells surrounding the somitocoel, filled with mesenchymal cells. A major part of somite cells is occupied by large nuclei, yolk platelets, and lipid droplets (Figure 1B). Since somitogenesis shows a cephalo-caudal gradient, in the anterior part of the embryo, differentiated somites with significant myotome are visible, whereas in the posterior part, somites remain still undifferentiated, made of cells forming a rosette-like structure (stage VIII) (Figure 1D). As somitogenesis proceeds (stage VIII), somites are subdivided into the dermomyotome, myotome, and sclerotome. The dermomyotome occupies the lateral surface of the myotome and consists of one layer of epithelial cells, whereas the ventro-medial situated sclerotome is mesenchymal. The morphological manifestation of myogenesis initiation is the elongation of somite wall cells in the medio-lateral direction, shown on the cross sections through the embryo (Figure 1C). The cells acquire a conical shape and then fuse to form two or three multinucleated muscle lamellae. The multinucleated muscle lamellae are perpendicularly orientated to the neural tube and notochord (stage X) (Figure 2).

**Figure 3:**
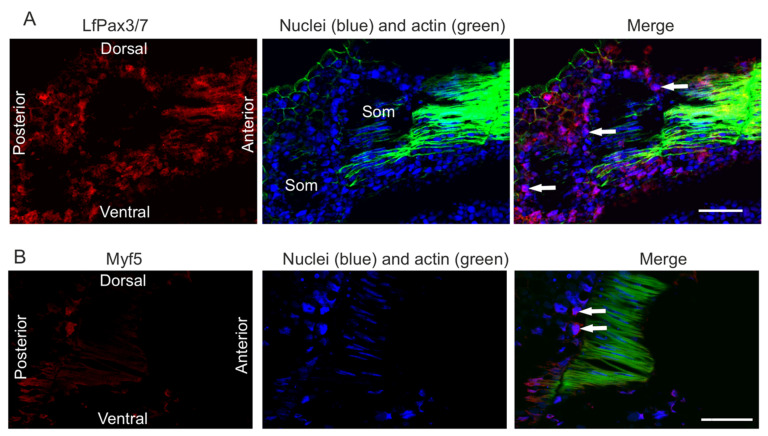
**LfPax3/7 and Myf5 presence in the river lamprey (*L.* *fluviatilis*) dermomyotome.** (**A**) Confocal cross section through the trunk muscles (stage XVII). LfPax3/7 (red), nuclei (blue), actin (green). (**B**) Confocal cross section of the trunk muscles (stage XXI). Myf5 (red), nuclei (blue), actin (green). The white arrows point out the LfPax3/7 and Myf5 signal in the nucleus. Somite (Som). Scale: 150 µm.

The ultrastructure revealed the presence of the first myofibrils beneath the sarcolemma at the peripheral part of each lamella. In the sarcoplasm, the lipid droplets, surrounded by glycogen granules, and yolk platelets are also visible and aggregated in terminal parts of muscle lamellae (Figure 2B, inset). On the cross sections through the embryo, a much higher yolk platelet content is visible in both terminal parts of each muscle lamella (Figure 2A). The first occurrence of muscle lamellae is observed in the vicinity of axial organs (neural tube and notochord), whereas in the lateral part of the myotome, mononucleated cells are still present (Figure 2A). These observations confirm that the myoblast fusion shows a medio-lateral gradient in each myotome (Figure 1E). Immunocytochemical analysis revealed the presence of LfPax3/7 and Myf5 proteins in the dermomyotome cells (Figure 3).

**Figure 4:**
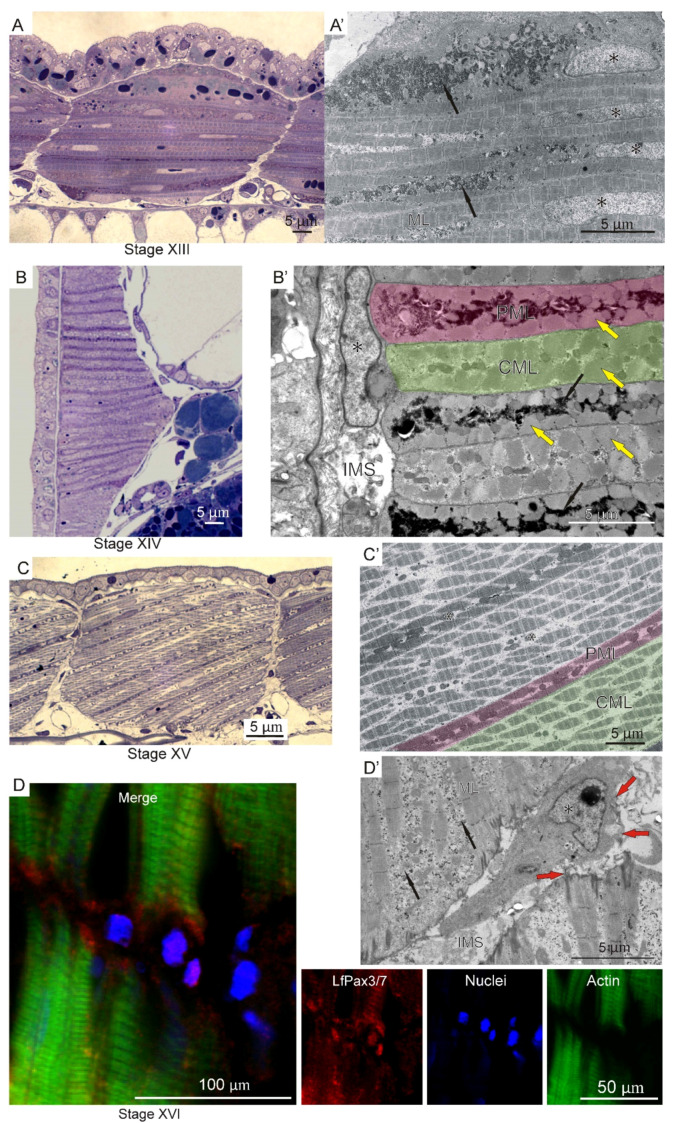
**Formation of central and parietal muscle lamellae in the river lamprey (*L.* *fluviatilis*).** Longitudinal (**A**,**C**) and cross sections (**B**) of trunk muscles stained with methylene blue. (**A’**–**D’**) Ultrastructure of skeletal muscles. (**D**) Confocal longitudinal section. LfPax3/7 (red), nuclei (blue), actin (green). Muscle lamellae (ML), glycogen granules (black, thin arrows), nuclei (*), parietal muscle lamellae (PML, colored in pink at (**B’**,**C’**)), central muscle lamellae (CML, colored in green at (**B’**,**C’**)), myofibrils (yellow arrows), intermyotomal space (IMS), collagen fibers (red arrows).

Initially, during myogenesis, the myotome is built of a homogeneous population of myogenic cells (muscle lamellae). No cells were observed in the intermyotomal space, which was confirmed by the analysis in light microscopy and TEM (stage XIII) (Figure 4A,A’). In the next stage of myogenesis (stage XIV) in the myotome, two types of muscle lamellae can be distinguished: the central type, surrounded by two parietal muscle lamellae (Figure 4B,B’). In the parietal muscle lamellae, the sarcoplasm is richer in glycogen granules compared to the central type (Figure 4B’). As myogenesis proceeds (stage XV), muscle lamellae become fully filled with myofibrils. Their nuclei are still located centrally. It is worth highlighting that central muscle lamellae are larger in diameter than the parietal type (Figure 4C,C’). At further stages of myogenesis (stage XVI), for the first time, mesenchymal cells are observed in the intermyotomal spaces. These cells are spindle-shaped and surrounded by collagen fibers, which confirms their mesenchymal character. Additionally, some of them are LfPax3/7-positive (Figure 4D,D’).

### 2.2. Adult Myogenesis

**Figure 5:**
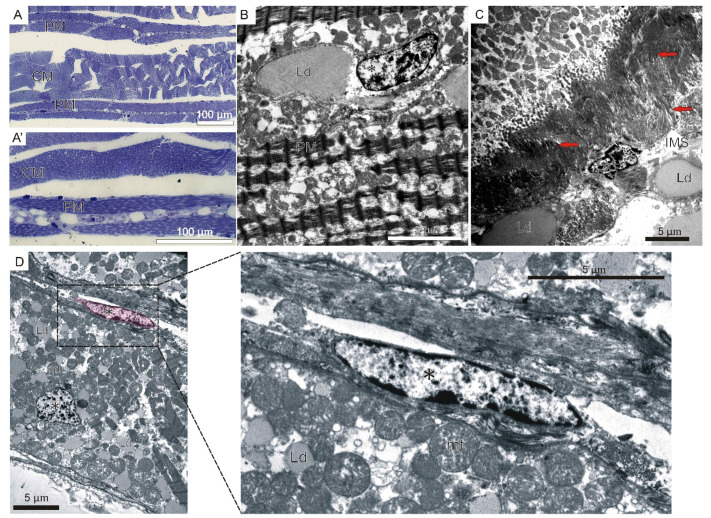
**Adult musculature of river lamprey (*L.* *fluviatilis*).** (**A**) Longitudinal sections of trunk muscles stained with methylene blue. Four central muscle fibers (CM), surrounded by groups of parietal muscle (PM) fibers. (**A’**) Zoomed view of CM and PM. (**B**) Ultrastructure of parietal muscles. (**C**) Ultrastructure of muscle–tendon junction. (**D**) Ultrastructure of muscle satellite-cell precursor (colored in pink) located along fiber. The proximity of the cell is shown in the enlarged photogram. Lipid droplets (Ld), nuclei (*), collagen fibers (red arrows), intermyotomal space (IMS), mitochondria (mt).

Based on the analysis of longitudinal and sagittal sections through the adult body, we observed that the myotomal muscles in the studied species are divided into two groups of muscles: muscles running in parallel and transversely to the long axis of the body. A characteristic feature of adult river lamprey musculature is the arrangement of muscles into myomeres (segments). The muscles in each segment are composed of four central muscle fibers and groups of parietal muscle fibers surrounding the central muscles on both sides (Figure 5A).

The central muscle fibers are fully filled with myofibrils with well-defined light and dark bands. Their nuclei assume a subsarcolemmal position (Figure 5A’). The parietal muscles are characterized by a different structure compared to the central muscles. The sarcoplasm of this group of muscles stains more strongly with methylene blue. Additionally, the central part of muscle fibers sarcoplasm is devoid of contractile apparatus. Moreover, numerous lipid droplets are observed in their sarcoplasm (Figure 5A’,B). The nuclei of the parietal fibers, similarly to the central fibers, occupy central and peripheral positions in the sarcoplasm (Figure 5B). The muscles directly contact with the area of the intermyotomal space, filled with collagen fibers and lipid droplets, creating muscle–tendon junctions (Figure 5C). TEM analysis revealed the presence of cells adjoining muscle fibers (Figure 5D).

## 3. Discussion

### 3.1. Somitogenesis

During river lamprey (*L. fluviatilis*) embryogenesis, the paraxial mesoderm undergoes segmentation, forming metameric units of cells, somites. Somites are built of a single layer of epithelial cells surrounding a somitocoel filled with mesenchymal cells. A similar pattern of somite architecture is observed in teleost fishes, e.g., *Danio rerio* [34,35], *Esox lucius* [13], *Rutilus frisii meidingeri* [36], and the bone-cartilaginous fish *Acipenser* *baeri* [11]. The Pax3- and Pax7-positive cells are characteristic of the dermomyotome among vertebrates, including teleost fishes (such as *D*. *rerio*, *Pterophyllum scalarae*, and *Coregonus* *lavaretus*), representatives of untiled amphibians (*Xenopus* *laevis)*, reptilians (*Natrix natrix*), and birds and mammals [14,15,16,26,37,38,39,40]. We confirmed that also in river lamprey the dermomyotome, like in other vertebrates, contains cells expressing Pax3/7.

The Pax3/7 protein belongs to a conservative cyclostome clade of orthologous sequences, including river lamprey *LfPax37* [41,42,43,44]. The conservative nature of Pax3/7 in lampreys was also observed in *Lampetra* *japonicum*, where *LjPax3/7-A* was found to be closely related to the jawed Pax3 and Pax7 genes, early regulators of muscle specification [45]. However, neither the amino acid sequence nor the phylogenetic analyses distinguish whether Pax3/7 is more closely related to *Pax3* or *Pax7*, present in jawed vertebrates [4,43]. Pax3/7 could have a broader mechanism of action, fulfilling both Pax3 and Pax7 functions.

In vertebrates, the Pax3 and Pax7 transcription factors lead to the activation of early myogenic regulatory factors (MRFs). Our analysis revealed, for the first time, the presence of the myogenic regulatory factor Myf5 in the lamprey dermomyotome. The Myf5 homologs can be found in species phylogenetically related to river lamprey, such as *Petromyzon marinus* (factor 5-like) and *Lethenteron camtschaticum* (myogenic regulatory factor MRF-A), indicating that Myf5 is also a conserved protein. Another MRF family member, a homolog of MyoD (LjMRF-A), was also found to be present in *L.* *japonica.* It was evidenced that in *L*. *japonica,* transcripts of LjMRF-A were located in the lateral somites at early stages, followed by expression at the dorsal and ventral edges of somites, overlapping with the expression of LjPax3/7-A genes [45]. In our studies, both Myf5 and LfPax3/7 staining is observed in the dermomyotome cells. These observations strongly confirm that, as in other vertebrates, the lamprey dermomyotome is a source of muscle progenitor cells.

Although lampreys are phylogenetically distant from jawed vertebrates, somite morphology, especially dermomyotome function, shows some similar features. Here we present evidence that may support the hypothesis that somitogenesis is a conservative process among all vertebrates. To confirm this hypothesis, future studies regarding the muscle differentiation, including the in vitro analysis of primary cell culture of embryonic myoblast, are needed.

### 3.2. Myogenesis

The presence of two- or three-nucleated muscle lamellae during river lamprey myogenesis is a unique feature. During the early steps of lamprey myogenesis, mononucleate myoblasts elongate and fuse to form structures resembling lamellae, in contrast to most vertebrates, where they form multinucleate muscle fibers. Multinuclear muscle lamellae are also present in the larval and adult musculature of amphioxus *Branchiostoma lanceolatum*, a representative of the Cephalochordata subphylum. However, contrary to our observations in river lamprey, *B*. *lanceolatum* muscle lamellae are mononuclear [46,47]. Additionally, multinucleated muscle lamellae were observed in other phylogenetically ancestral fish species, such as sturgeon (*Acipenser* *baeri*) and lungfish (*Neoceratodus forsteri*). Similarly to river lamprey, in sturgeon and lungfish, muscle lamellae were observed only during larval stages. Adult musculature of *L*. *fluviatilis*, *A*. *baeri*, and *N*. *forsteri*, like in other vertebrates, is built of cylindrical multinucleated muscle fibers [11,12,37]. Whereas in sturgeon and lungfish the mechanism of muscle lamellae conversion into cylindrical muscle fibers has been well studied, in the lamprey this process is still unknown. The main reason is the long lamprey larval period (3–7 years), which makes obtaining individuals during the metamorphosis period extremely difficult.

In river lamprey, the first muscle lamellae are observed in the vicinity of axial organs (neural tube and notochord), whereas in the lateral part of the myotome, the mononucleated cells are still observed. The axial organs stimulate myogenesis in the lateral part of the myotome, and therefore myogenesis shows a mediolateral gradient. In lampreys, *Hedgehog* (*Hh*) is expressed in the midline of the embryo. As in zebrafish, homologs of *patched* (a receptor for Hh signaling) and *prdm1* (a target of Hh signaling) are expressed in adaxial regions of the lamprey somite. An *engrailed* (*eng*) homolog, a signal responsible for differentiation into muscle pioneer cells, is also expressed in the lamprey somite [48].

As our study shows, initially, during river lamprey myogenesis, the myotomes are built of a homogeneous population of myogenic cells (muscle lamellae). Our observations confirm the absence of any cells in intermyotomal spaces at this early stage of development. The mesenchymal Pax3/7 positive cells appear in these regions at the further stages of myogenesis. Our findings are consistent with the observations made in fishes such as *C*. *lavaretus* and *P*. *scalarae*, where Pax3-positive cells were also observed in the intermyotomal spaces and subsequently in the myotomes between the myotubes. In studied fishes, those cells differentiate into satellite cells, closely adhering to muscle fibers [14,16].

### 3.3. Adult Muscle Fibers

The characteristic muscle structure pattern observed in the river lamprey larvae consists of a central surrounded by two parietal muscle lamellae. In adults, the parietal muscles are characterized by a different structure compared to the central muscles. Light microscopy analysis showed that in the latter group the sarcoplasm in the central part of muscle fibers is devoid of contractile apparatus. On the other hand, the presence of numerous lipid droplets is a characteristic feature of parietal muscle fibers. This morphological characteristic corresponds to the structure of the slow fibers, whereas central muscles resemble white muscle fibers present in the higher vertebrates’ musculature. Indeed, lamprey parietal muscles are classified as slow contracting fibers using assays showing the oxidative enzymes and myosin ATPase distribution and activity in *L.* *japonica* [49]. These results were also confirmed using Sudan black B (SBB) and succinic dehydrogenase (SDH) staining [50]. In contrast, central muscles show histochemical characteristics similar to fast contracting white muscle fibers. Ikeda et al., (2013) [51] compared heavy chains of myosin (MYHs) expressed in fast skeletal muscles among vertebrates with their lamprey counterparts.

### 3.4. Ancestral Features in River Lamprey Myogenesis

The adult musculature in all vertebrates is built of multinuclear muscle fibers. A comparative analysis of the early stages of myotome myogenesis in Chordata indicates that the myogenic process in this phylum follows an evolutionarily conservative course. The effect of myogenesis, the mature muscles, is obtained through different strategies. In amphioxus (*B*. *lanceolatum*), the myotome is composed of more than one thousand thin lamellae, stacked together. Throughout the life cycle, each myotomal muscle cell (lamella) remains mononucleated [46,47,52]. In the early stages of myogenesis of river lamprey, we detected the presence of multinucleated muscle lamellae. Our observations are consistent with investigations made in other lampreys (e.g., *L*. *japonica*) and fish representatives (such as sturgeon and lungfish), where muscle lamellae were also present during larval stages [11,12,53].

We conclude that the presence of muscle lamellae can be treated as a plesiomorphic (ancestral) feature of Chordata myogenesis, whereas the presence of multinucleated muscle fibers is an evolutionary success of vertebrates. It is interesting that lampreys and other ancestral representatives of fishes—sturgeons and lungfish—share the same pattern of early muscle differentiation. Animals of this group, including lampreys, undergo the same pattern of holoblastic cleavage, which is unique in the case of fish, because all teleost fish undergo meroblastic cleavage. We conclude that lamprey myogenesis shares some similar features with both ancestral (presence of, e.g., muscle lamellae) and more advanced chordates (e.g., multinuclearity of muscle fibers). Which of the transcription factors controlling myogenesis in these groups of animals is responsible for the formation of muscle lamellae remains to be investigated and clarified.

## 4. Materials and Methods

### 4.1. Animals and Sampling Sites

The river lamprey (*L. fluviatilis*) was obtained in late October from the Vistula River, near Tczew, Poland. After capture, lampreys were transported to the Center for Aquaculture and Ecological Engineering in Olsztyn in accordance with the rules applying to transport of live animals. Animals were placed in two fish spawning tanks [54]. Water temperature in the tanks was kept at 6.5 ± 0.5 °C. At the beginning of April, the temperature was increased to 11 ± 0.5 °C, to stimulate gonad maturation. From then on, spawners were inspected every two days to assess lampreys’ reproductive maturity. The eggs and sperm were collected from sexually mature individuals. The eggs were fertilized with sperm from 3–5 male individuals. After fertilization, eggs were incubated separately in the Weiss apparatus at 12 ± 0.5 °C. The percentage of fertilization success was analyzed. For further analysis, the larvae from the group with the highest fertilization rate (over 95%) were taken. During the embryo and larvae incubation, samples consisting of several dozen eggs were taken every day and fixed. The process of sampling lasted until the larvae resorbed the yolk sac. Developmental stages were estimated using developmental tables of *L. fluviatilis* published by Richardson et al., 2010 [55].

### 4.2. Aminoacidic Sequence Analysis of LfPax3/7 and Myf5

**Figure 6:**
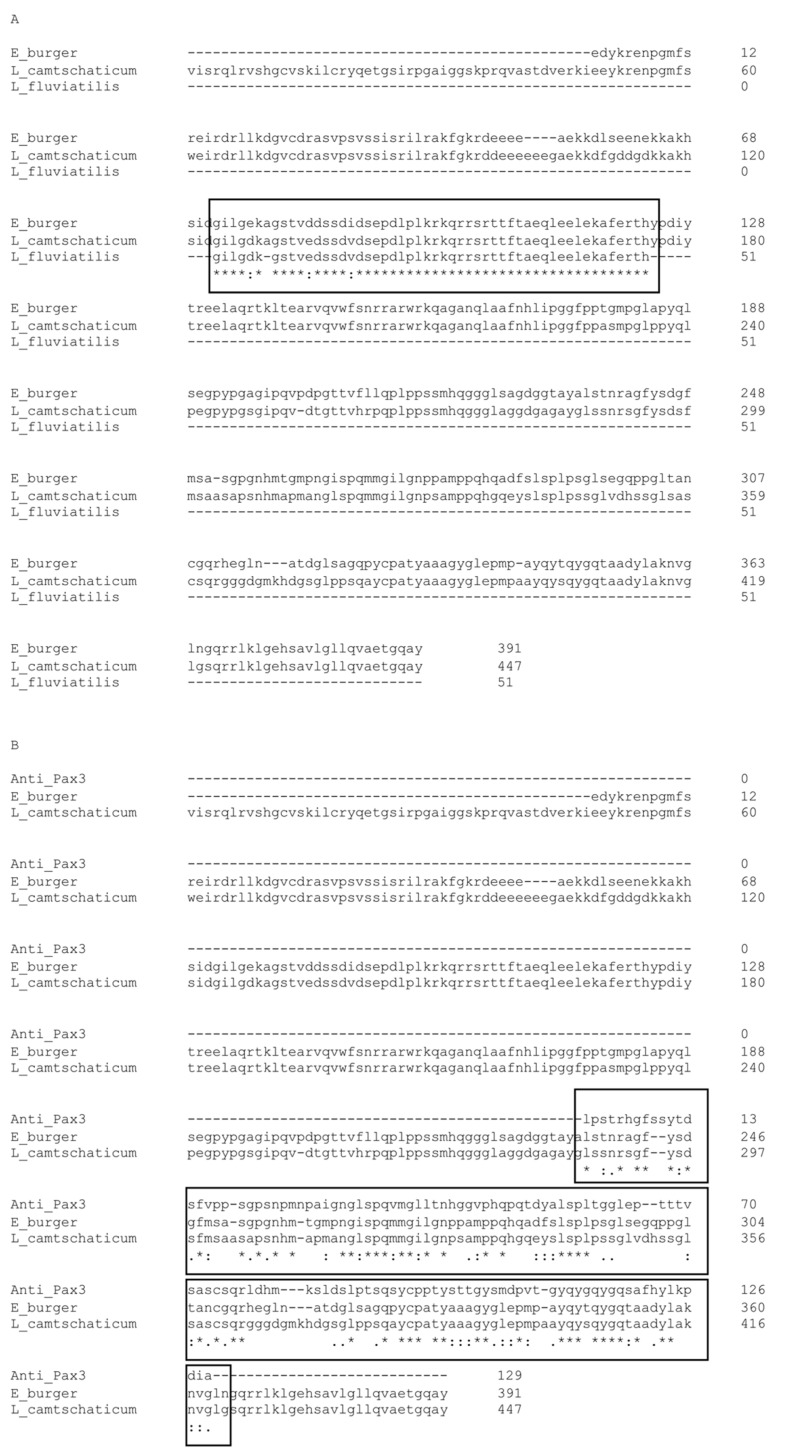
**The conserved structure of river lamprey (*Lampetra fluviatilis*) protein *LfPax3/7*.** (**A**) The aminoacidic sequences alignment of *L. fluviatilis LfPax37* (GenBank: AAY90105.1, *partial*), *Eptatretus burger* paired-box protein 3/7 (GenBank: BAG11537.1), and *Lethenteron camtschaticum* paired-domain transcription factor Pax3/7-A (GenBank: ADP37890.1). The black frame shows the homological fragments. (**B**) The mouse monoclonal anti-Pax3 (Developmental Studies Hybridoma Bank, cat. # 528426) antibody epitope shows a high degree of homology with both *LfPax37* orthologs present in *E. burger* and *L. camtschaticum* (indicated with a black frame). An * (asterisk) indicates positions that have a single, fully conserved residue; A: (colon) indicates conservation between groups of strongly similar properties, equivalent to scoring > 0.5 in the Gonnet PAM 250 matrix; A: (period) indicates conservation between groups of weakly similar properties, equivalent to scoring =< 0.5 and > 0 in the Gonnet PAM 250 matrix. The amino acid sequence alignment was performed using Clustal Omega, version 1.2.2, currently maintained at by Des Higgins, Fabian Sievers, David Dineen, and Andreas Wilm, Conway Institute UCD Dublin, Dublin, Ireland [56].

The analysis of available data shows the conservative nature of amino acid sequences of the LfPax3/7 protein (Figure 6). The Pax3/7 protein belongs to a conservative cyclostome clade of orthologous sequences, including *L. fluviatilis* LfPax37, *Eptatretus burger* paired-box protein 3/7, and *Lethenteron camtschaticum* paired-domain transcription factor Pax3/7-A [41,43,44] (Figure 6A). The known, partial sequence fragment of LfPax37 shows a high degree of homology with both orthologues (Figure 6A, black frame). Since there are no commercially available, specific antibodies against lamprey LfPax37, we used the mouse monoclonal anti-Pax3 (Developmental Studies Hybridoma Bank, cat. # 528426) antibody to visualize the Pax3/7 lamprey ortholog (protein LfPax37). The antibody epitope shows a high degree of homology with both LfPax37 orthologs from *E. burger* and *L. camtschaticum* (Figure 6B).

**Figure 7:**
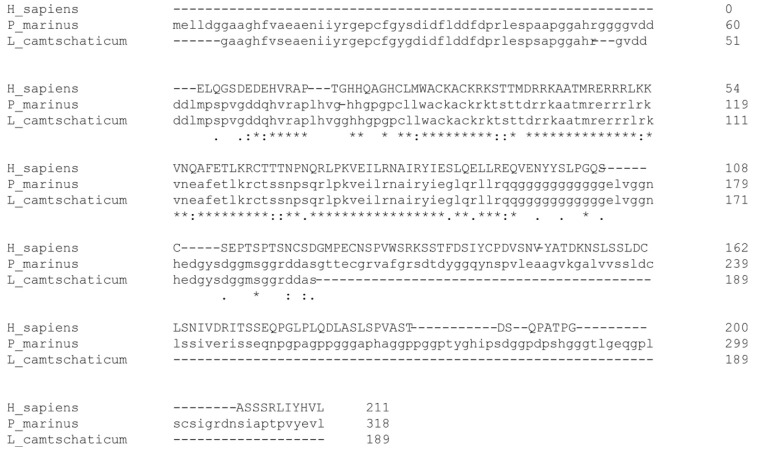
**The conserved structure of river lamprey (*Lampetra fluviatilis*) myogenic factor Myf5.** The amino acid sequences alignment of human Myf5 sequence (Gene ID: 4617) and lampreys orthologs: *Petromyzon marinus* factor 5-like (NCBI Reference Sequence: XP_032827047.1) and *Lethenteron camtschaticum* myogenic regulatory factor MRF-A (GenBank: ADP37889.1). An * (asterisk) indicates positions that have a single, fully conserved residue; A: (colon) indicates conservation between groups of strongly similar properties, equivalent to scoring > 0.5 in the Gonnet PAM 250 matrix; A: (period) indicates conservation between groups of weakly similar properties, equivalent to scoring =< 0.5 and > 0 in the Gonnet PAM 250 matrix. The amino acid sequence alignment was performed using Clustal Omega, version 1.2.2, currently maintained at by Des Higgins, Fabian Sievers, David Dineen, and Andre-as Wilm, Conway Institute UCD Dublin, Dublin, Ireland [56].

The analysis of data available in genomic databases shows that the aminoacidic sequence of Myf5 is evolutionary conservative. We used the BLAST® (Bethesda, MD, USA) National Center for Biotechnology Information bioinformatics tool to search for sequences similar to human Myf5 (Gene ID: 4617) among lampreys [57]. The analysis shows the presence of proteins highly similar to myogenic factor 5 (Myf5) in the species closely related to *L. fluviatilis.* Those orthologs include *Petromyzon marinus* factor 5-like (NCBI Reference Sequence: XP_032827047.1) and *Lethenteron camtschaticum* myogenic regulatory factor MRF-A (GenBank: ADP37889.1) (Figure 7). Such conservation in the protein sequence among lampreys indicates that Myf5 could be also conserved in *L. fluviatilis*. Indeed, in our research, we were able to detect this protein using the MYF5 antibody produced with the use of a synthesized peptide derived from human MYF5 (GeneTex, cat. # GTX87110).

### 4.3. Light and Transmission Electron Microscopy

For light and electron microscopic examination, the embryos or larvae were fixed in 2.5% glutaraldehyde in 0.1 M phosphate buffer pH 7.2 for 24 h at 4 °C. The material was rinsed 3 times with phosphate buffer and was postfixed for 2 h in a 1:1 mixture of osmium tetroxide-potassium ferricyanide (OsO_4_-K_3_Fe (CN)_6_). Following rinsing in the phosphate buffer, the material was dehydrated, first in a graded alcohol series and then in acetone. Then, it was embedded in epoxy resin Epon 812 (Sigma-Aldrich, St. Louis, MI, USA) [58]. The Epon blocks were sectioned on a Leica Ultracut UCT (Leica, Wetzlar, Germany). Semithin sections (0.6 μm) were collected on glass slides and stained with methylene blue (in a 1% borax solution). The stained sections were examined under an Olympus BX60 light microscope (Olympus, Hamburg, Germany). The ultrathin sections were collected on 200-mesh copper grids and were stained with uranyl acetate and lead citrate according to the standard protocol [59], before being examined under the transmission electron microscope JEM-1011 (JEOL, Tokyo, Japan).

### 4.4. Immunofluorescence Analysis

The material was prepared, and immunofluorescence reactions were carried out on tissue cryosections as previously described [58]. The following primary antibodies were used: mouse monoclonal anti-Pax3 (Developmental Studies Hybridoma Bank) at a dilution of 1:50 in phosphate buffer saline with 0.1% Tween-20 (PBST); rabbit polyclonal anti-Myf5 (GeneTex, Hsinchu, Taiwan) at a dilution of 1:200 in PBST. Additionally, donkey anti-mouse IgG Cy3 conjugated, and donkey anti-rabbit IgG Cy5 conjugated (Jackson ImmunoResearch, West Grove, PA, USA) at a dilution of 1:100 in PBST secondary antibodies were used. The standard control of primary antibody specificity was performed by staining tissues also with secondary antibodies only (see the Supplementary Figure S1). The DNA was stained with 4,6-diamidino-2-phenylindole (DAPI; 0.2 μg/mL in PBS). The actin cytoskeleton was visualized using Alexa Fluor 488- or Alexa Fluor 546-conjugated phalloidin (Molecular Probes, Eugene, OR, USA) at a dilution of 1:80 in PBS. For keeping consistent coloring through the results section, the LfPax3/7 and Myf5 signal is always shown in red, while phalloidin staining is green. For the imaging, an Olympus FluoView FV1000 confocal laser scanning microscope (Olympus) was used.

## Supplementary Materials

The following supporting information can be downloaded at: https://www.mdpi.com/article/10.3390/ijms23158595/s1.
