# Supplementary material for: Unique Features of River Lamprey (Lampetra fluviatilis) Myogenesis
Source: Int J Mol Sci. 2022 Aug 2;23(15):8595. doi: 10.3390/ijms23158595 (PMC9368804; doi:10.3390/ijms23158595)
Supplement: Supplementary file 1 [file ijms-23-08595-s001.zip › ijms-1782043-supplementary.pdf]

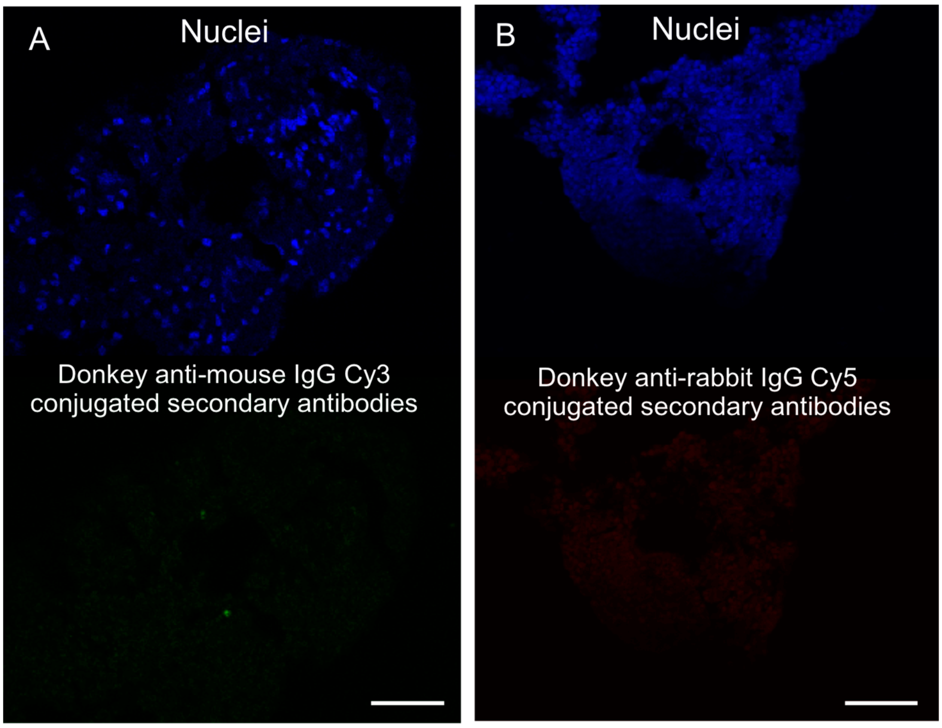

Figure S1. The control of primary antibody specificity

The DNA was stained with 4,6-diamidino-2-phenylindole (DAPI; 0.2 μg/ml in PBS). No primary antibodies were used. Instead, tissues were stained using donkey anti-mouse IgG Cy3 conjugated (A), and donkey anti-rabbit IgG Cy5 conjugated (B) (Jackson ImmunoResearch, West Grove, PA, US) secondary antibodies at a dilution of 1:100 in PBST. Scale 25 μm.
